# The effect of clothes on blood pressure measurement

**DOI:** 10.12669/pjms.331.11811

**Published:** 2017

**Authors:** Nurcan Ertug, Tugba Cakal, Seyda Busra Ozturk, Muhammet Verim

**Affiliations:** 1 Nurcan Ertug, RN PhD. Assistant Professor, School of Nursing, Ufuk University, Ankara, Turkey; 2 Tugba Cakal, BSN. Nurse, Turgut Ozal University Hospital, Ankara, Turkey; 3 Şeyda Busra Ozturk, BSN. Nurse, Turgut Ozal University Hospital, Ankara, Turkey; 4 Muhammet Verim, BSN. Nurse, Turgut Ozal University Hospital, Ankara, Turkey

**Keywords:** Blood pressure, Blood pressure measurement, Clothes, Health professionals, Sphygmomanometer

## Abstract

**Objective:**

To determine the effect of clothes on blood pressure measurement.

**Methods:**

One group pretest-posttest design was used in this study. The study consisted of 162 undergraduate students studying nursing and physiotherapy at a university in Ankara, Turkey. Blood pressure was measured over the sleeve and below a rolled-up sleeve with a mercury-filled column sphygmomanometer. All blood pressure measurements were performed on the right arm during morning hours by the same nurse. Each participant’s height, weight and clothing thickness were measured.

**Results:**

The mean age of the participants was 20.71. The median systolic blood pressure values were 110.07 mmHg over the sleeve and 110.37 mmHg below the rolled-up sleeve. There were no statistically significant differences between measurements taken over the sleeve and below a rolled-up sleeve (*p*=0.222). The median diastolic blood pressure values were 69.56 mmHg over the sleeve and 69.59 mmHg below the rolled-up sleeve. There were no statistically significant differences between measurements taken over the sleeve and below a rolled-up sleeve (*p*=0.572).

**Conclusion:**

It was found that clothes have no statistically significant effect on systolic/diastolic blood pressure measurements. Measuring blood pressure over a sleeve may save time.

## INTRODUCTION

Blood pressure measurement is one of the most common practices performed by health care professionals in everyday practice. Since blood pressure is 1 of the 4 vital signs, its accurate measurement is crucial. In order to make proper decisions regarding diagnosis and treatment, blood pressure measurement should be accurate. Tomlinson^1^ pointed out that concern exists among healthcare professionals about patient care decisions being made on the basis of inaccurate blood pressure measurements and the resulting unexpected negative outcomes. If measurements are not performed accurately and reliably, there is a potential for significant harm and excessive costs. An incorrect blood pressure measurement of 5 mmHg higher than actual blood pressure can result in the false diagnosis of hypertension in as many as 27 million people in the U.S. If one considers that one year of hypertension treatment has a cost of 1000 U.S. dollars, the annual cost of these false diagnoses could result in a waste of $27 billion.^2^

We observed that health professionals generally measure the blood pressure below patients’ rolled-up sleeves. If tight sleeves cannot be rolled up, they commonly do not want to spend the time to remove patients’ clothes, so they measure blood pressure over the sleeve. Instead of examining whether measuring blood pressure over a sleeve is accurate, healthcare professionals often focus on completing the task. In various textbooks, bare arm measurement was reported as necessary but the reason why was not usually given.^3^-^6^ However, measuring a person’s blood pressure over a sleeve is more practical than measuring it below a rolled-up sleeve or on a bare arm. Measuring over a sleeve saves time, especially in emergency situations, and helps maintain patient privacy. However, there are few data about whether the presence of clothes affects blood pressure measurements in normotensive people.

We searched Scopus, Medline and CINAHL databases with no date restrictions in English using search terms “blood pressure”, “clothes” and “sleeve”. Our review of the literature found several studies for the effect of clothes on blood pressure measurement. Each study included both male and female subjects. Most of these studies included normotensive and hypertensive patients; featured wide age, body mass index (BMI), and clothing thickness ranges; and used automatic sphygmomanometers.^7^-^10^ In only one study, a mercury-filled column sphygmomanometer was used for the blood pressure measurements.^11^ The studies also used various measurement numbers and positions. Blood pressure was measured over a sleeve and on the bare arm in 2 studies^11^,^12^; over a sleeve, below a rolled-up sleeve, and on the bare arm in 2 studies^7^,^10^; and over a sleeve and below rolled-up sleeve in one study.^9^ While it is reported that clothing does not have any effect on blood pressure measurement, it is evident that study populations in these groups were heterogeneous with respect to age, sex, BMI, and hypertension/normotension. In contrast with these studies, our study accounts for these confounding factors by using a homogeneous study population. The aim of this study was to determine the effect of clothes on blood pressure measurements in normotensive young females.

## METHODS

One group pretest-posttest design was used in this study. The inclusion criterion was to be young females at least 18 years of age. The exclusion criteria were subjects who: (1) had a diagnosis of hypertension, (2) had BMI≥30, (3) had ingested caffeinated beverages and cigarettes within 30 minutes before the blood pressure measurements and (4) had an empty bladder. These criteria of inclusion/exclusion were chosen due to the fact that blood pressure is affected by factors such as age, sex, BMI, smoking, caffeine intake, and body position.^4^,^13^-^19^

This study was conducted between February and May 2013 with a total number of 185 female undergraduate students studying nursing and physiotherapy at a university in Ankara, Turkey.

The students were invited to participate in this study and168 students volunteered to take part in the study. We excluded six students who had BMI≥30. However, none of the participants had been previously diagnosed with hypertension. Consequently, 162 participants were included in this study (participation rate, 87.5%).

The data were collected at the university’s a professional skills laboratory. Each participant was interviewed about their health history. Each participant’s weight, height, and clothing thickness were measured and recorded in the morning. Blood pressure was measured in accordance with an international guideline.^20^ The participants were requested to empty their bladders and refrain from ingesting caffeinated beverages and smoking cigarettes for 30 minutes before the measurements as these factors may cause a temporary increase in blood pressure.^21^-^24^

The participants generally dressed in clothes such as shirts, blouses, and sweaters. They were seated for at least five minutes before measurement of blood pressure. They were instructed to lean back in the seat, place their feet on the ground, and position their right arm at heart level. The participants were requested to refrain from moving and talking during the blood pressure measurement.

All blood pressure measurements were performed on the right arm during morning hours by the same nurse. Blood pressure was measured 2 times for each participant using the same sequence: over the sleeve and below a rolled-up sleeve. The nurse waited 2 minutes between measurements to allow adequate circulation to return.

During the planning and execution of this study, it was reported in updated guidelines that the mercury-filled column sphygmomanometer is the gold standard for measurement of blood pressure.^20^,^24^ Therefore, a mercury-filled column sphygmomanometer with an adult size cuff (16×30 cm) was used to measure blood pressure. Accuracy of this device is ±2 mmHg. The sphygmomanometer was calibrated at the beginning of the study.

Each participant’s clothing thickness was measured using a skinfold caliper. Participants’ height and weight were measured using a measuring tape and weighing scale, respectively, after which BMI was calculated. Socio-demographic data were collected via a questionnaire including age, smoking status, chronic diseases, and medication usage.

This study was approved by Fatih University Ethics Committee and conducted in compliance with the Declaration of Helsinki. The purpose and design of the study were explained to the students. Confidentiality and anonymity were guaranteed. Verbal information was given and written informed consent was obtained from all participants. Written permissions were obtained from the head of institutions.

Data were analyzed using Statistical Package for Social Sciences version 16.0 software (SPSS Inc., Chicago, IL, USA). Distribution normality was assessed using the Kolmogorov–Smirnov test, which revealed a skewed distribution. The differences among each participant’s two blood pressure measurements were analyzed using the Wilcoxon test. The Mann–Whitney U test was used to compare differences between blood pressure measurements and smoking status, chronic disease, or medication usage while the Kruskal–Wallis test was conducted to compare multiple groups (such as BMI and clothing thickness). Statistical significance was defined as *p*<0.05.

## RESULTS

All of the participants were Caucasian. The mean age of the students was 20.71 (SD=2.16) ranging from 18 to 36 years old. The mean BMI was 22.08 and the mean clothing thickness was 1.00 mm. The other characteristics of participants are summarized in Table-I.

**Table-I:** Characteristics of participants.

Characteristics	Participants (n=162)
Age years Mean (SD)	20.71 (2.16)
***Smoking status***	
Yes	11 (6.8)
No	151 (93.2)
***Chronic diseases***	
Yes	14 (8.6)
Cardiopulmonary diseases	4 (2.5)
Endocrine diseases	4 (2.5)
Neurologic diseases	4 (2.5)
Gastrointestinal diseases	1 (0.6)
Hematologic diseases	1 (0.6)
No	148 (91.4)
***Medication usage***	
Yes	9 (5.6)
Endocrine medications	4 (2.5)
Cardiopulmonary medications	2 (1.2)
Neurologic medications	1 (0.6)
Gastrointestinal medications	1 (0.6)
Hematologic medications	1 (0.6)
No	153 (94.4)
***Body Mass Index***	
Mean (SD)	22.08 (2.99)
<18.49	20 (12.3)
18.50–24.99	112 (69.1)
25–29.99	30 (18.6)
***Clothing thickness***	
Mean (SD)	1.00 (0.46)
≤ 0.5 mm	56 (34.6)
0.6–1.0 mm	63 (38.9)
1.1–1.5 mm	29 (17.9)
1.6–2.0 mm	14 (8.6)

The mean and standard deviation values of systolic–diastolic blood pressure measurements in two positions is shown in Table-II. The median systolic blood pressure (SBP) values were 110.07 (SD=11.31) mmHg over the sleeve and 110.37 (SD=10.94) mmHg below the rolled-up sleeve. There were no statistically significant differences between measurements taken over the sleeve and below a rolled-up sleeve (*p*=0.222). The median diastolic blood pressure (DBP) values were 69.56 (SD=8.95) mmHg over the sleeve and 69.59 (SD=9.03) mmHg below the rolled-up sleeve. There were no statistically significant differences between measurements taken over the sleeve and below a rolled-up sleeve (*p*=0.572).

**Table-II:** Comparisons of blood pressure measurements in two different positions

Blood pressure measurements	Over the sleeve	Below a rolled-up sleeve	Z test*	p value
SBP^†^ Mean (SD)	110.07 (11.31)	110.37 (10.94)	-1.222	0.222
DBP^‡^ Mean (SD)	69.56 (8.95)	69.59 (9.03)	-0.565	0.572

* : Wilcoxon test;

† : Systolic blood pressure;

‡ : Diastolic blood pressure.

The effect of participants’ socio-demographic characteristics on SBP/DBP measurements for both over the sleeve and bellow a rolled-up sleeve are shown in Table-III. As seen in this table, there were no statistical differences between over the sleeve and below a rolled-up sleeve SBP/DBP measurements in terms of the participants’ smoking status, chronic disease, medication usage, BMI, and clothing thickness (*p*>0.05).

**Table-III:** Over sleeve and below rolled-up sleeve systolic/diastolic blood pressure measurements in terms of the participants’ socio-demographic characteristics.

Variables	n (%)	Over the sleeve SBP^a^ Mean (SD)	U test/x^2^ p value	Below rolled-up sleeve SBP Mean (SD)	U test/x^2^ p value	Over the sleeve DBP^b^ Mean (SD)	U test/x^2^ p value	Below rolled-up sleeve DBP Mean (SD)	U test/x^2^ p value
***Smoking status***								
*Yes*	11 (6.8)	107.82 (16.38)	709.500	107.82 (15.37)	663.500	68.36 (10.42)	718.000	68.18 (11.29)	734.500
*No*	151 (93.2)	110.24 10.91)	0.419^c^	110.56 (10.59)	0.265^c^	69.65 (8.86)	0.452^c^	69.70 (8.88)	0.521^c^
***Chronic diseases***								
*Yes*	14 (8.6)	107.57 (9.48)	823.500	107.71 (8.18)	906.000	68.00 (8.22)	759.500	67.57 (8.60)	758.500
*No*	148 (91.4)	110.31 (11.46)	0.204^c^	110.62 (11.16)	0.437^c^	69.71 (9.02)	0.098^c^	69.78 (9.07)	0.097^c^
***Medication usage***								
*Yes*	9 (5.6)	107.53 (8.07)	559.000	107.65 (6.86)	618.500	65.29 (7.84)	412.000	66.59 (7.57)	453.000
*No*	153 (94.4)	110.37 (11.61)	0.343^c^	110.69 (11.30)	0.608^c^	70.06 (8.96)	0.062^c^	69.94 (9.14)	0.084^c^
***Body Mass Index***									
*<18.49*	20 (12.3)	106.90 (11.67)	3.529	107.30 (11.39)	2.892	68.30 (9.31)	0.196	67.80 (9.42)	0.875
*18.50–24.99*	112 (69.1)	109.93 (11.10)	0.171^d^	110.34 (10.73)	0.236^d^	69.78 (9.07)	0.906^d^	70.04 (8.97)	0.646^d^
*25–29.99*	30 (18.6)	112.73 (11.60)		112.53 (11.28)		69.60 (8.45)		69.13 (9.13)	
***Clothing thickness***									
*≤ 0.5 mm*	56 (34.6)	109.07 (11.49)	1.364	109.57 (10.89)	1.272	70.39 (8.94)	1.742	70.54 (9.24)	2.344
*0.6–1.0 mm*	63 (38.9)	110.38 (11.51)		110.13 (10.88)		68.86 (9.19)		68.76 (8.77)	
*1.1–1.5 mm*	29 (17.9)	111.72 (11.00)	0.714^d^	111.86 (11.10)	0.736^d^	68.90 (9.66)	0.628^d^	68.83 (9.87)	0.504^d^
*1.6–2.0 mm*	14 (8.6)	109.29 (11.00)		111.57 (11.90)		70.79 (6.47)		71.14 (7.79)	

a Systolic blood pressure,

b Diastolic blood pressure,

c Mann-Whitney U test,

d Kruskal-Wallis test.

## DISCUSSION

In this study, the effect of clothes on blood pressure measurements was evaluated in normotensive female subjects with a mercury-filled column sphygmomanometer. No statistical differences were determined between SBP/DBP measurements taken over the sleeve and below a rolled-up sleeve. These data are supported by the findings of previous studies.^7^,^9^-^12^

Ki et al.^7^ and Kahan et al.^10^ measured BP in normotensive/hypertensive male/female patients over a wide age range using an automatic sphygmomanometer. In those studies, the BP of each patient was measured three times: over a sleeve, below a rolled-up sleeve, and on a bare arm. The clothing thickness of the vast majority of patients (83.7%) was < 2 mm^7^ and the mean clothing thickness was 1.7 mm^10^. Neither study reported statistically significant differences among the three BP measurements. These findings are in line with our study.

Thien et al.^12^ measured the normotensive/hypertensive male/female patients’ blood pressure over the sleeve and on the bare arm using an oscillometric device. They did not find differences in blood pressure between over the sleeve and bare arms. This study supports to our findings.

Liebl et al.^9^ and reported that BP measurements taken over a sleeve do not differ significantly from those taken on bare arms. That study included normotensive/hypertensive and male/female patients and used both manual auscultatory and automatic sphygmomanometers. The mean variables were as follows: age, 45.5 years; BMI, 23.4; and clothing thickness (of 77.1% of patients), < 1 mm. Pinar et al.^11^ studied 258 male/female hypertensive patients (clothing thickness, ≤ 2 mm; BMI range, 20–41) with a mercury-filled column sphygmomanometer. SBP and DBP were measured over a sleeve and on a bare arm. These researchers found no statistical differences in BP between 2 groups.

The blood pressure measurement devices and positions as well as subject sexes, hypertension status, BMI, and clothing thicknesses differed between the current study and previous studies. However, despite these methodological differences, our results support those of previous studies regarding the effect of clothes on blood pressure measurements.

### Study limitations

This study has two limitations. First, the nurse who performed the blood pressure measurements was not blinded because of the nature of the study. Second, since the clothing thickness was not more than two mm, no comparisons can be made regarding the effect of thicker clothing on blood pressure measurement.

## CONCLUSIONS

No statistically significant effect of clothes on SBP/DBP measurements was found. As such blood pressure measurement can be taken without the patient removing their clothing or rolling up a sleeve. Additionally, there was no statistical difference detected between SBP/DBP measurements and participants’ smoking status, chronic disease, medication usage, BMI, or clothing thickness. Over-the-sleeve blood pressure measurements are recommended for normotensive female with clothing up to 2 mm thick. Since the thickness of clothing was 2 mm or less in this study, future studies should investigate whether or not thicker clothing has any effect on measurements.
